# A New International Nursing Course

**Published:** 1921-06-11

**Authors:** 


### A New International Nursing Course.

The League of Red Cross Societies announces an interesting extension of the Public Health course arranged for nurses of various nations in connection with the University of London. The first course, as our readers know, is now in progress at the King's College for Women branch of the University, and nineteen nurses, representing eighteen countries, entered for it. A new course, we learn, is being organised at Bedford College for Women, also a part of the University of London, and will commence on October 7. Nurses from Japan, China, and New Zealand have already entered their names. Red Cross Societies all over the world belonging to the League have been requested to offer scholarships to enable Red Cross nurses to take this valuable course, which will run concurrently with that again arranged to take place at King's College for Women. The amount of each scholarship is $1,000, to cover tuition and living expenses for ten months; travelling expenses to and from London will also be granted. Candidates must be between twenty-three and thirty years, must show evidence of continuous education up to eighteen years of age, must possess a diploma certificate of nursing conforming to the highest nursing standard in their own country, and must have sufficient knowledge of English to follow lectures.

The League of Red Cross Societies is doing good work in thus preparing Public Health nurses from various nations to be centres of light and leading in their own countries. Under the able conduct of Miss Alice Fitzgerald, Director of the Department of Nursing to the League, the Division of Nursing has assumed a foremost place in Red Cross activities. By special request from any country to the Department investigations will be made on the spot by expert members of the staff with a view to either improving the nursing service or inaugurating a nursing centre should there be nothing to start with. It is desired to secure the services of several well-trained public health nurses to act as field secretaries, visit the different countries represented by the London students, and assist in the removal of obstacles to nursing progress. Already Miss Fitzgerald has under the supervision of her department the London University courses and the nursing staff of child-welfare units in Slovakia and Roumania, while she acts as adviser to the Canadian Nursing Unit in Bucarest, and the committee of the nurse-training school in Belgrade. Two British nurses are employed by the L.O.R.C.S. and ten others are working under its auspices. Additional nursing personnel is not required at the Geneva headquarters, the League being purely a central consulting body to aid Red Cross societies in developing their work.

